# Psychopathological Differences Between Self-Injurious Behaviors and Suicide Attempts in Adolescents

**DOI:** 10.5152/eurasianjmed.2023.21287

**Published:** 2023-02-01

**Authors:** Hicran Doğru

**Affiliations:** 1 Department of Child and Adolescent Psychiatry, Faculty of Medicine, Atatürk University, Erzurum, Turkey.

**Keywords:** Adolescent, non-suicidal self-injury, suicide attempt, self-harm

## Abstract

**Objective:**

Suicidal attempts and self-injurious behavior are major public health concerns, and they are strong predictors of death in youths worldwide. Given the risk of death, there is an urgent need to understand the differences and identify effective interventions. This study aimed to investigate the relationship between the predictors associated with non-suicidal self-injury and suicide attempts among adolescents.

**Materials and Methods:**

The study recruited a total of 61 adolescents aged 12-18 years, with suicide attempts (n = 32) and non-suicidal self-injury (n = 29). Turgay Disruptive Behavioral Disorders Screening and Rating Scale-Parent form, Rosenberg Self-esteem Scale, and Beck Anxiety and Beck Depression Inventory assessment scales were applied. All participants were interviewed with the structured clinical interview for Diagnostic and Statistical Manual of Mental Disorders, fourth edition.

**Results:**

The adolescents with the suicide attempts were found to have lower self-esteem, higher depression, inattention and hyperactivity-impulsivity scores than the group with non-suicidal self-injury. Higher inattention scores and rural residency were positively and significantly associated with suicide attempts, adjusting for other discrimination types (odds ratio = 1.250, 95% CI = 1.024-1.526; odds ratio = 4.656, 95% CI = 1.157-18.735).

**Conclusion:**

This study shows that some clinical psychiatric factors may be helpful in distinguishing adolescents with suicide attempts from adolescents with non-suicidal self-injury. Future research is needed to determine the predictive role of these variables in distinguishing suicidal attempts from self-injurious behavior.

**Main Points**

- Self-injurious behaviors and suicide attempts often co-occur in adolescents.
- The difference between self-injurious behaviors and suicide attempts has yet to be elucidated.
- Adolescents with suicide attempts may have lower self-esteem, higher depression scores, higher inattention, and hyperactivity/impulsivity (H/I) subscale scores than adolescents with self-injurious behaviors.
- Environmental (rural residency) and cognitive factors (higher inattention scores) may pose a risk for suicide attempts in adolescents.

## Introduction

Self-injurious behavior, whether suicidal or not, is a serious public health problem affecting adolescents and young adults globally.^1^ Mechanisms associated with self-regulation such as coping (cognitive and behavioral response processes) and emotion regulation (emotional response processes) are thought to underlie this behavior among adolescents.^2^ Frequent or various types of injuries are associated with more suicidal behavior compared with infrequent and less varied types of injuries.^3^ Injury behaviors can be divided into intentional and unintentional injuries. Intentional injuries are divided into 3 groups as suicide, non-suicidal self-injury (NSSI), or violent attacks.^4^

Although research supports the distinction between suicide attempts (SAs) and NSSI, the overlap between the 2 phenomena has been identified in clinical populations in up to 70%.^5^ Both phenomena were found to be associated with high levels of depression, suicidal ideation, and hopelessness. In addition, those who attempted suicide had higher scores on measures of anxiety, depression, and suicidal ideation than those with NSSI.^6^ Also, a suicidal attempt has more serious consequences than NSSI, and the risk of suicide for adolescents with NSSI is also considerably higher. Being able to identify the differences between the 2 phenomena might help the clinician to define adolescents who would attempt suicide.

Suicide, one of the leading causes of death worldwide, has even more worrying consequences for young people. It is estimated to be the second cause of death among young people aged 10-24.^7^ Suicide risk has been associated with sociodemographic variables such as gender, age, marital status, economic status, and educational status.^8^ Other clinical features associated with SAs are as follows: tobacco and alcohol use, exposure to traumatic stressful events, such as abuse; physical illness, somatic symptoms, anxiety; some psychological factors, such as hopelessness, impulsivity, low self-esteem, loneliness, anger, and appetite loss.^8–12^

Adolescence is a risky period in terms of self-injurious behavior due to difficulties in coping with stress and in regulating emotions.^13^ Some of the stressors and sociocultural factors mentioned above for both behavior of NSSI and SAs may rise self-regulation difficulties in adolescents and further increase the risk of self-injury.^13^ Also, considering the relationship between the above-mentioned risky predictors, it can be thought that some disorders such as depression, anxiety disorders, and attention-deficit/hyperactivity disorder (ADHD) may increase these risky behaviors in youth. Indeed, researchers have studied psychiatric disorders with self-injurious behavior in youths and have reported very high prevalence figures. In a review that included data from 24 countries, it was found that psychiatric disorders were identified in 81.2% of adolescents with self-injurious behavior.^14^ The most common disorders according to the data were depression, anxiety, and alcohol abuse, respectively, as well as ADHD and conduct disorder among youths.

In summary, adolescents with a psychiatric disorder show an increased risk for SAs and NSSI. Besides, there is high evidence that some sociodemographic variables also contribute to these behaviors. Identifying variables that mediate between SAs and NSSI may further the comprehension of underlying mechanisms and lead the clinical practice. Here, we aimed to investigate the relationship between SAs and NSSI by evaluating sociodemographic and diagnostic predictors by examining the effects of ADHD symptomatology, anxiety-depression scores, and self-esteem levels.

## Materials and Methods

### Participants

The participants consisted of adolescents who were admitted to the emergency department with the complaint of NSSI or SA between 2016 and 2018 and then evaluated in a “face-to-face” child and adolescent psychiatry outpatient clinic in a training and research hospital. In the specified time, 76 cases with SAs or NSSI were consulted by the department of child and adolescent psychiatry, but a total of 61 adolescents (NSSI = 29, SAs = 32) were included in the study. Fifteen cases were excluded because 2 cases had autism, 1 case had a brain tumor, 10 cases refused to interview, and 2 cases died after suicide.

### Procedures

Adolescents, who were admitted to the emergency department with complaints of suicide or NSSI, were invited to undergo further examination (structured clinical interview and adolescent/parent scales). Written informed consent was obtained from the parents of the adolescents participating in the study.

Inclusion time started in 2016 and we excluded those with prior NSSI or SAs. The Kiddie Schedule for Affective Disorders and Schizophrenia–Present and Lifetime Version for Diagnostic and Statistical Manual of Mental Disorders, fourth edition (K-SADS-PL DSM-IV) was used to obtain information on mental health disorders. Patients diagnosed with psychosis and substance addiction were also included in the study. However, those with organic problems were not included in the study, and they were triaged into a separate unit. We excluded cases of self-injury behaviors related to a diagnosis of autism or intellectual disability and accidental self-injury behaviors. In addition, adolescents and their parents who did not know Turkish were not included in the study.

In addition, only the first admission data were used for the cases who were admitted to the emergency department more than once during the study period. All study procedures have been approved by the Institutional Review Board. Ethical approval was obtained from the Ethics Committee of Atatürk University, School of Medicine (2018/19-199).

### Measurements

The Kiddle Schedule for Affective Disorders and Schizophrenia for School-Age Children Present and Lifetime: This form is a semi-structured interview form that evaluates the current and past psychopathology of children and adolescents according to DSM-IV.^15^

Beck Depression Inventory: This is a 21-item self-report scale that assesses the current severity of depression with a total score ranging from 0 to 63.^16^

Beck Anxiety Inventory: This is a 21-item self-report scale that assesses the current severity of anxiety with a total score ranging from 0 to 63.^17^

The Rosenberg Self-Esteem Scale: This scale is a 10-question scale that defines an individual’s general assessment of self-worth. It includes 5 positive words and 5 negative words and is scored using 4 response options ranging from strongly agree to strongly disagree.^18^ The level of self-esteem in this test can be summarized as follows: 0 to 1 “high,” 2 to 4 “medium,” and 5 to 6 “low.”

Turgay DSM-IV-Based Disruptive Behavior Disorders Child and Adolescent Rating and Screening Scale-parent form: This scale is widely used to determine ADHD subtypes, severity and disruptive behavioral problems based on DSM-IV diagnostic criteria. This parent-reported scale was adapted to Turkish by Ercan.^19^ This is a four-point Likert-type scale including: inattention (9 items), hyperactivity-impulsivity (9 items), opposition/defiance (8 items), and 15 items for conduct disorder. Symptoms are scored on a 0-3-point Likert-type scale by assigning an estimate of severity for each symptom. Higher scores indicate more severe problems. In this study, the total score of each subgroup was used.

### Data Analysis

Descriptive statistics were reported for the basic sociodemographic variables. Also, relations between some variables were evaluated using the chi-square test and the independent sample *t*-test, as appropriate. Shapiro–Wilk test was used to determine the normal distribution. The Pearson’s and Spearman’s correlation analyses were used, as appropriate. Logistic regression analysis (forward selection) was performed to determine independent risk factors for SAs, including variables that were statistically significant in the univariate analysis. Statistical analyses were performed with Statistical Package for the Social Sciences software version 20 (IBM SPSS Corp.; Armonk, NY, USA). Statistical significance was defined as *P* < .05.

## Results

In total, 61 adolescents aged 12-17 years were included in the study. Fifty-one (83.6%) were females and 10 (16.4%) were males. The median age was 15 years and the interquartile range (IQR) was 15-16 years. Thirty-two adolescents (52.5%) had SAs. There was no significant gender difference between SAs and NSSI, but adolescents were predominantly female in both groups (84.4% vs. 82.8%, χ^2^ = 0.29, *P* = .865). Table 1 presents the sociodemographic features and the comparison between adolescents with SAs and NSSI.

According to the answers of the cases to the question of why they exhibit this behavior, the conditions causing these behaviors were, respectively, unhappiness (n = 10), trauma history (n = 9), anger management problem (n = 8), hopelessness (n = 4), and psychotic delusions (n = 1) in the SAs group. In the NSSI group, the most common reasons were anger management problems (n = 13), trauma history (n = 7), hopelessness (n = 5),unhappiness (n = 3), and psychotic delusions (n = 1), respectively. Also, related to trauma history, 5 of the SAs group and 2 of NSSI group reported having been sexually abused. However, there was no significant difference between the groups (*P* = .269).

The rates of smoking, alcohol abuse, and substance abuse were higher in adolescents with SAs, but there was no significant difference between the groups (*P* = .609, *P* = .307, and *P* = .674). In both groups, there was a family history of having a parent with aggressive behavior, and the rates were high but there was no significant difference between the 2 groups (SA: 62.5% and NSSI: 72.4%). The rates of pregnancy stress history were 58.6%(n=17) in the NSSI group and 78.1%(n = 25) in the SAs group. There was also a significant difference between the 2 groups in terms of residency (*P* = .017). The rates of intrafamilial conflict between the groups were also as follows: SAs: 59.5% and NSSI group: 40.5%. However, there was no difference between the 2 groups. There was no significant difference between the 2 groups in terms of age, ethnicity, socioeconomic status, education level, school success, pregnancy stress, physical illness history, and family history of mental illness (*P* > .05).

Both groups were compared in terms of BDI, BAI, and RSES. In adolescents with SAs, depression scores, and inattentive and hyperactivity/impulsivity (H/I) subscale scores were higher and self-esteem was lower than in the group with NSSI (Table 2.).

Table 3 presents the correlation analyses between scale scores and some variables. There was a positive correlation between the year of self-injury (the total duration of years after self-injurious behavior has started) and age (*r_p_* = 0.271, *P* = .035). There was also a positive correlation between inattentive and hyperactivity/impulsivity subscale scores (*r_p_* = 0.470, *P* = .01). Besides, there was a positive correlation between RSES and BAI scores (*r_p_* = 0.374, *P* = 0.01), RSES, and BDI scores (*r_p_* = 0.644, *P* = .01).

Binary logistic regression analysis was conducted to predict SAs vs. NSSI. The Nagelkerke R^2^ was 0.414. This model comparing suicide attempts with NSSI found high inattentive scores (odds ratio = 1.25, 95% CI = 1.024-1.526, *P* = .028) and rural residency (odds ratio = 4.656, 95% CI = 1.157-18.735, *P* = .030) to be contributing factors (Table 4).

## Discussion

Adolescents with SAs showed higher depression scores, higher inattentive and hyperactivity/impulsivity symptom scores, and lower self-esteem compared to the NSSI, whereas all scale scores combined with other predictors, inattentive symptom scores remained consistent in contributing to the difference between the groups. These results support the findings of previous research showing the effect of ADHD symptomology on suicidal behavior.^21^

Studies have found a relationship between ADHD and risk factors for suicide and self-injurious behaviors.^22^ The severity of ADHD symptomatology has also been shown to have a significant relationship with these behaviors.^23^ In this study, inattention and hyperactivity/impulsivity scores were found to be associated with suicide attempts. As is known, suicide attempt poses a greater risk of death than self-injurious behaviors. Given the association of ADHD symptomatology with a range of other high-risk behaviors, it is not surprising that the SAs group had higher ADHD scores than the NSSI group. However, hyperactivity/impulsivity scores were not an adjustable predictor of SAs according to the regression model. The fact that the hyperactivity/impulsivity symptom scores remained significant in the pairwise comparison but not in the regression model may suggest its mediating role rather than a direct effect on SAs. Also, this result may be due to the small sample size.

The groups were similar in terms of age, gender, education, and economic status. Interestingly, the adolescents were predominantly female in both groups. Considering that the participants were recruited according to the admissions to the emergency room, it can be thought that females in both groups needed more emergency service support than males. Also, the rate of youth living in rural areas was higher in the SAs group than in the NSSI group. Studies have shown that adolescents living in rural areas are almost twice as likely to die by suicide as those living in urban areas.^24^ Factors contributing to adolescent suicides in rural areas include mental health, labor shortages, poverty, and increased access to lethal tools.^24^ Considering the conditions of the region where our study was conducted, the possible explanation for this relationship was the limited mental health services in rural areas. The higher symptom scores of disorders and probable less access to care may explain the difference in the group with SAs.

In line with the literature, depressive and anxiety symptoms were quite high in both groups.^20^ In addition, depressive symptom scores were higher in the SAs group than in the NSSI group, even though the depression levels of both groups were severe according to the BDI. A higher depression score posed a risk for SAs; nevertheless, it was not an adjustable predictor of SAs according to our logistic regression model. This may suggest a role for multifactor combined risks rather than a single factor effect for SAs in adolescents.

Adolescents in both groups reported high rates of unhappiness, hopelessness, trauma history, anger management problem, self-cutting, and family conflict; also, 5 of the SA group and 2 of the NSSI group had sexual abuse history. It is known that such risky psychosocial and psychiatric factors cause behaviors such as NSSI and/or SAs in adolescents.^25,26^ This may suggest not only that psychiatric disorders pose a significant risk for SAs and NSSI but also that the nature of this risk occurs after adverse life events. Therefore, it is not surprising that NSSI might accompany SAs.^27^ Further, due to the multiple risk factors underlying self-harming behaviors, it isn't easy to differentiate NSSI from SA quantitatively.^25^ In this study, 65.6% of the SAs group were found to be accompanied by NSSI behaviors. So, high comorbidity may also cause complex differences between SAs and NSSI.

In sum, suicide is the second leading cause of death in adolescence. Understanding that adolescents will attempt suicide has important implications for suicide prevention and early intervention. Therefore, identifying contemporaneous risk predictors for SAs is critical. Although there was a common etiology between SAs and NSSI groups, we found some significant differences between the 2 groups. Our findings increase the need for greater awareness of ADHD and depression symptomatology, self-esteem levels, and rural residency in adolescents with suicidal attempts.

One limitation of our study included the cross-sectional nature of the study which limits providing a clear inference about the directionality of the relationship between sociodemographic and clinical data and SAs in self-injurious adolescents. The other limitation of the study is a small sample size that makes the findings less generalizable to other groups of adolescents with suicidal attempts. The results need to be confirmed by longitudinal studies.

Self-injurious behavior and SA which are strong predictors of death are common in adolescents worldwide. Our study shows that social–clinical measures (higher depression scores and lower self-esteem), environmental (rural residency), and cognitive measures (higher inattention scores) differentiate adolescents with and without a history of SAs. Further studies are needed to confirm whether the findings identified in this study differentiate those with SAs from those with NSSI and predict subsequent SAs in a larger sample of adolescents. Also, our study provides clues for clinicians about individual–social–environmental interventions that may contribute to the prevention of suicide in adolescents and highlights the need for close monitoring of adolescents with SAs and NSSI.

## Figures and Tables

**Table 1.** Demographic Characteristics

	**Non-suicidal Self-Injury **	**Suicide**	***P***
	**n = 29 (%)**	**n = 32 (%)**	
Age mean (STD)	15 (1.27)	15.5 (1.24)	.592
Sex			
Male	5 (17.2)	5 (15.6)	.865
Female	24 (82.8)	27 (84.4)	
Residency			
Rural	6 (27.3)	16 (50)	**.017**
Urban	23 (79.3)	16 (50)	
Family type			
Nuclear	23 (79.3)	20 (62.5)	.151
Single parent	6 (20.7)	12 (37.5)	
Economic status			
Low	13 (44.8)	17 (53.1)	.578
Middle	12 (41.4)	13 (40.6)	
High	4 (13.8)	2 (6.2)	
Current education status			
Secondary school	5 (17.2)	3 (9.4)	.371
High school	21 (72.4)	22 (68.8)	
Dropped out of school	3 (10.3)	7 (21.9)	
School success			
Good	12 (41.4)	12 (37.5)	.757
Poor	17 (58.6)	20 (62.5%)	
Pregnancy stress			
Yes	17 (58.6)	25 (78.1)	.100
No	12 (41.4)	7 (21.9)	
Number of self-mutilative acts			
1	2 (6.9)	8 (25)	**.05**
More than 1	27 (93.1)	24 (75)	
Watching video about self-injury			
Yes	20 (69)	13 (40.6)	**.027**
No	9 (31)	19 (59.4)	
Self-cutting			
Yes	29 (100)	21 (65.6)	**.00**
No	0	11 (34.4)	

The bold value of 0.017 represents Pearson Chi-Square test.

**Table 2.** Comparison of Groups’ Scale Scores

	**Non-suicidal Self-Injury (n = 29)**	**Suicide (n = 32)**	***P***
	**Mean ± SD**	**Mean ± SD**	
BDI score	30.4 ± 9.8	36.1 ±11.5	**.044**
BAI score	25.1 ±11.8	27.4 ± 13.3	.488
RSES score	2.1 ± 0.9	2.8 ± 1.1	**.01**
Inattention subscale score	6.7 ± 3.8	9.9 ± 4.1	**.003**
Hyperactivity/impulsivity (H/I) subscale score	8.9 ± 4.2	11.4 ± 4.2	**.027**
Associated symptoms score	9.0 ± 4.4	8.3 ± 4.1	.537

RSES, The Rosenberg Self-Esteem Scale score; BDI, Beck Depression Inventory; BAI, Beck Anxiety Inventory; SD, standard deviation.

Significant bold value represents 2-tailed significance.

**Table 3.** Bivariate Correlation Analysis Between Scale Scores

	**1**	**2**	**3**	**4**	**5**	**6**	**7**	**8**
1. Age	-							
2. Duration of self-injury	0.271*							
3. Inattentive subscale score	0.053	0.025						
4. Hyperactivity/impulsivity (H/I) subscale score	0.157	0.004	0.470**					
5. Associated symptoms score	0.206	0.173	−0.166	0.216				
6. BDI score	−0.019	−0.086	0.065	0.165	−0.120			
7. BAI score	0.072	0.053	−0.030	−0.060	0.025	0.464**		
8. RSES	−0.090	0.016	0.060	0.136	−0.111	0.644**	0.374**	-

*r*, Spearman’s correlation coefficient, *correlation is significant at the 0.05 level, **correlation is significant at the 0.01 level.

RSES, The Rosenberg Self-Esteem Scale score; BDI, Beck Depression Inventory; BAI, Beck Anxiety Inventory.

Significant bold value represents 2-tailed significance. *P* = 0.035, correlation= 0.271.

**Table 4.** Predictors of Suicidal Attempts in the Cohort

	***B***	**SE**	**Wald**	**Sig.**	**OR**	**95% CI for EXP (B)**	
						**Lower**	**Upper**
Inattentive subscale score	0.223	0.102	4.818	**0.028**	1.250	1.024	1.526
Hyperactivity/impulsivity (H/I) subscale score	0.048	0.087	0.297	0.58	1.049	0.884	1.245
Associated symptoms score	−0.041	0.087	0.220	0.63	0.960	0.810	1.138
RSES score	0.780	0.421	3.433	0.06	2.182	0.956	4.983
BAI score	0.007	0.030	0.047	0.83	1.007	0.949	1.068
BDI score	0.005	0.040	0.018	0.89	1.005	0.929	1.089
Residency	1.538	0.710	4.690	**0.03**	4.656	1.157	18.735

OR, odds ratio; SE, standard error; RSES, The Rosenberg Self-Esteem Scale score; BDI, Beck Depression Inventory; BAI, Beck Anxiety Inventory.

Reference categories: NSSI (for group), urban (for residency).

Significant bold value represents 2-tailed significance.
